# All-Polymer Printed Low-Cost Regenerative Nerve Cuff Electrodes

**DOI:** 10.3389/fbioe.2021.615218

**Published:** 2021-02-10

**Authors:** Laura M. Ferrari, Bruno Rodríguez-Meana, Alberto Bonisoli, Annarita Cutrone, Silvestro Micera, Xavier Navarro, Francesco Greco, Jaume del Valle

**Affiliations:** ^1^Center for Micro-BioRobotics @SSSA, Istituto Italiano di Tecnologia, Pontedera, Italy; ^2^The BioRobotics Institute and Department of Excellence in Robotics and AI, Scuola Superiore Sant'Anna, Pontedera, Italy; ^3^Université Côte d'Azur, INRIA, Sophia Antipolis, France; ^4^Department of Cell Biology, Physiology and Immunology, Institute of Neurosciences, Universitat Autònoma de Barcelona, and CIBERNED, Bellaterra, Spain; ^5^Bertarelli Foundation Chair in Translational NeuroEngineering, Center for Neuroprosthetics and Institute of Bioengineering, Ecole Polytechnique Federale de Lausanne, Lausanne, Switzerland; ^6^Institute of Solid State Physics, NAWI Graz, Graz University of Technology, Graz, Austria; ^7^Department of Life Science and Medical Bioscience, Graduate School of Advanced Science and Engineering, Waseda University, Tokyo, Japan

**Keywords:** regenerative cuff electrodes, low-cost fabrication, inkjet printing, wrinkling, organic bioelectronics, PEDOT:PSS, peripheral nerve interfaces

## Abstract

Neural regeneration after lesions is still limited by several factors and new technologies are developed to address this issue. Here, we present and test in animal models a new regenerative nerve cuff electrode (RnCE). It is based on a novel low-cost fabrication strategy, called “Print and Shrink”, which combines the inkjet printing of a conducting polymer with a heat-shrinkable polymer substrate for the development of a bioelectronic interface. This method allows to produce miniaturized regenerative cuff electrodes without the use of cleanroom facilities and vacuum based deposition methods, thus highly reducing the production costs. To fully proof the electrodes performance *in vivo* we assessed functional recovery and adequacy to support axonal regeneration after section of rat sciatic nerves and repair with RnCE. We investigated the possibility to stimulate the nerve to activate different muscles, both in acute and chronic scenarios. Three months after implantation, RnCEs were able to stimulate regenerated motor axons and induce a muscular response. The capability to produce fully-transparent nerve interfaces provided with polymeric microelectrodes through a cost-effective manufacturing process is an unexplored approach in neuroprosthesis field. Our findings pave the way to the development of new and more usable technologies for nerve regeneration and neuromodulation.

## Introduction

Injuries to the peripheral nervous system (PNS) result in the partial or total loss of the motor, sensory and autonomic functions of the body part innervated by the lesioned nerve. The quality of life of people who suffer from these conditions is significantly reduced and major social consequences are payed in terms of health-care (Rosberg et al., 2005). When the whole nerve is transected and the two nerve ends cannot be surgically rejoined, an autologous nerve graft is commonly interposed in clinical practice to support axons regeneration. Nerve conduits have been extensively reported as a clinical alternative to the autograft repair (Deumens et al., 2010). However, even with surgical repair, axonal regeneration may fail or be insufficient to allow functional reinnervation of targets and recovery of all motor and sensory functions. Prostheses and exoskeletons are therapeutic strategies used to improve patients' quality of life after severe nerve injuries or limb amputations. For the application of neuroprosthesis, peripheral nerve interfaces (PNIs) are a key element as they provide a link between the nervous system and the mechanical device (del Valle and Navarro, 2013).

Typically, PNIs are produced through state-of-the-art microfabrication and micromachining processes. Standard lithographic techniques, characterized by expensive high vacuum and high temperature processes, are commonly and efficiently used for the last 20 years (Stieglitz and Meyer, 1999; Boretius et al., 2010). Together with the drawbacks of cleanroom processes, some of the traditional metallic materials adopted (e.g., Au, Pt) have limitations in interfacing the soft biological tissues (Bellamkonda et al., 2012). In the last decade, organic electronic materials (mainly conducting polymers and semiconductors) have shown their potential in applications where electronics interfaces biology. Given their soft mechanical properties and mixed conductivity (ionic and electronic) (Rivnay et al., 2013), they proved to be an optimal bioelectronic interface, as already demonstrated in a variety of applications (Berggren and Richter-Dahlfors, 2007; Khodagholy et al., 2013; Simon et al., 2016). Organic materials represent the new frontier for PNIs development, since they can enhance signal quality, biocompatibility and chronic reliability (Bettinger, 2018). Particularly, organic materials have shown an increased charge injection capacity compared to standard metallic materials (Green et al., 2013), thus enabling the miniaturization of electrode arrays (Bettinger, 2018).

One of the benefits of adopting organic electronic materials is that they can be suited for printing. Inkjet printing is a large-scale and low-cost production process. It is a non-contact additive manufacturing method that operates in ambient conditions, at low temperatures. This allows the adoption of flexible substrates, with limited stability at high temperature, such as thermoplastic foils often exploited in flexible/printed electronics (Wong and Salleo, 2009; Caironi and Noh, 2015). The technique is suited for the deposition of various functional materials, as conductive polymers (CPs), organic semiconductors, polymers used for dielectric/passivation, which are solution processable (Berggren et al., 2007; Elschner et al., 2010). However, because of intrinsic limitations in resolution, inkjet printing cannot reach the miniaturization level required in some applications and addressable by cleanroom processing. Typically inkjet printing of features with lateral size smaller than ~100 μm can be challenging, at least with commercially available facilities. On the other hand, there is a growing interest in adopting self-assembling methods for surface patterning, especially on a large area. Surface wrinkling is a self-assembling phenomenon which has been proposed as a rapid and convenient method for surface patterning (Genzer and Groenewold, 2006; Rodríguez-Hernández, 2015). It allows for tunable texturing with quasi periodic topographic motifs (with features size ranging from tens-hundreds of nm up to mm scale) over very large area (up to several m^2^). Wrinkled surfaces of various materials have been successfully tested in various applications, such as stretchable electronics, sensors and cell cultures, among others (Chen et al., 2011; Greco et al., 2012). One of the possible approaches to obtain surface wrinkling relies on the use of heat-shrink thermoplastic substrates for the 3D patterning of thin films deposited onto them. By thermally inducing the in-plane shrinkage of the substrate, the top-deposited thin film buckles, resulting in a conformal wrinkled skin (Greco et al., 2013; Bonisoli et al., 2017). Concurrently, the heat-shrinking provides a suitable method for miniaturization (down to e.g., 10% of original size) of any pattern (i.e., circuit, electrode), while retaining the pristine large surface area (Gabardo et al., 2017; Chan et al., 2018). In this way, the charge injection capacity of the electrode per unit area can be increased, which is an attractive feature especially for stimulating electrodes (Green and Abidian, 2015). Moreover, electrodes miniaturization has positive effects on improving signal resolution during neural recording (Lacour et al., 2016).

Here we propose a novel non-conventional microfabrication technique to produce low-cost all-polymer PNIs. The produced design, named regenerative nerve cuff electrode (RnCE), consists in a flexible plastic tube embedding conducting polymer microelectrodes facing the lumen. Miniaturized 3D textured electrodes are produced by the combination of inkjet printing and shrink-induced surface wrinkling (Print and Shrink). To this aim, a heat-shrinkable polyolefin (PO) wrap film is used as low-cost, optically transparent and flexible substrate. Heat-shrink PO films are commonly adopted in food packaging and have been proposed as biocompatible substrates for cell culturing (Chen et al., 2011; Lew et al., 2011; Sharma et al., 2011), as well as molds for PDMS substrates in similar applications (Nguyen et al., 2009; Chen et al., 2014; Shum et al., 2017). The materials here adopted for the electrodes patterning are poly(3,4-ethylenedioxythiophene):poly(styrene sulfonate) (PEDOT:PSS) and SU-8. PEDOT:PSS, the most used conducting polymer in bioelectronics, is commercially available as waterborne dispersion and it has shown excellent chemical-physical stability and biocompatibility (Groenendaal et al., 2000; Bernards et al., 2008; Malhotra and Ali, 2017). Biocompatibility and stability of various printable PEDOT:PSS formulations have been investigated, revealing the crucial role of thermal annealing and additives (Stríteský et al., 2018). In implantable devices, PEDOT:PSS has shown to induce lower foreign body reaction (FBR) over other polymers (Cellot et al., 2016). Moreover, the use of CPs as coatings for neural electrodes was reported to lower the impedance at the electrode-tissue interface as compared to the bare metal electrode (Asplund et al., 2010), thus enhancing the charge transfer and downplaying the electrical insulation effect due to the encapsulation of the implant. SU-8, an epoxy resin-based dielectric, is used for electrical passivation, given its biocompatibility (Nemani et al., 2013) and the widespread use in neural probes applications (Cho et al., 2008; Altuna et al., 2013; Márton et al., 2020).

One of the main challenges for regenerative PNIs is the production of non-obtrusive interfaces, defined as transparent interfaces, still retaining a good degree of stimulation selectivity. Previous works remarked the effort in the assessment of transparent electrodes, as the regenerative multielectrode interface (REMI) (Garde et al., 2009), the regenerative scaffold electrode (RSE) (Clements et al., 2012) and more recently the double-aisle regenerative electrode (Delgado-Martínez et al., 2017), comprising a silicone tube with a polyimide foil interposed in between and gold microelectrodes located on the two opposing sides of the foil. The latter was developed by exploiting the same manufacturing process used for intraneural PNIs, such as the thin film longitudinal intrafascicular electrode (tf-LIFE) (Lago et al., 2007) or the transversal intrafascicular multielectrode (TIME) (Boretius et al., 2010), and its design allowed unobstructed nerve regeneration, further enabling an interfacing with the axons. Despite the plurality of advantages of the design, the techniques used for the manufacturing of the above-mentioned devices are very expensive and require a cleanroom environment.

In this article we describe the RnCEs low-cost fabrication process and their performance as a regenerative nerve guide and PNI *in vivo*. Firstly, we assessed the RnCEs design as permissive for axonal regeneration across a nerve gap by using passive (i.e., without polymeric microelectrodes) PEDOT:PSS coated PO tubes (PPP tubes). Once the adequacy for axonal regeneration has been confirmed, the active RnCEs, with patterned polymeric microelectrodes, was implanted and tested *in vivo* for nerve stimulation. Functional analysis, comprising electrophysiological tests and sensory recovery, was performed. After 90 days, the implanted RnCEs were tested for their ability to stimulate the regenerated nerve axons and their selectivity assessed in relation to transparency.

## Materials and Methods

### Regenerative Nerve Cuff Electrode (RnCE) Fabrication

**Figure 1:**
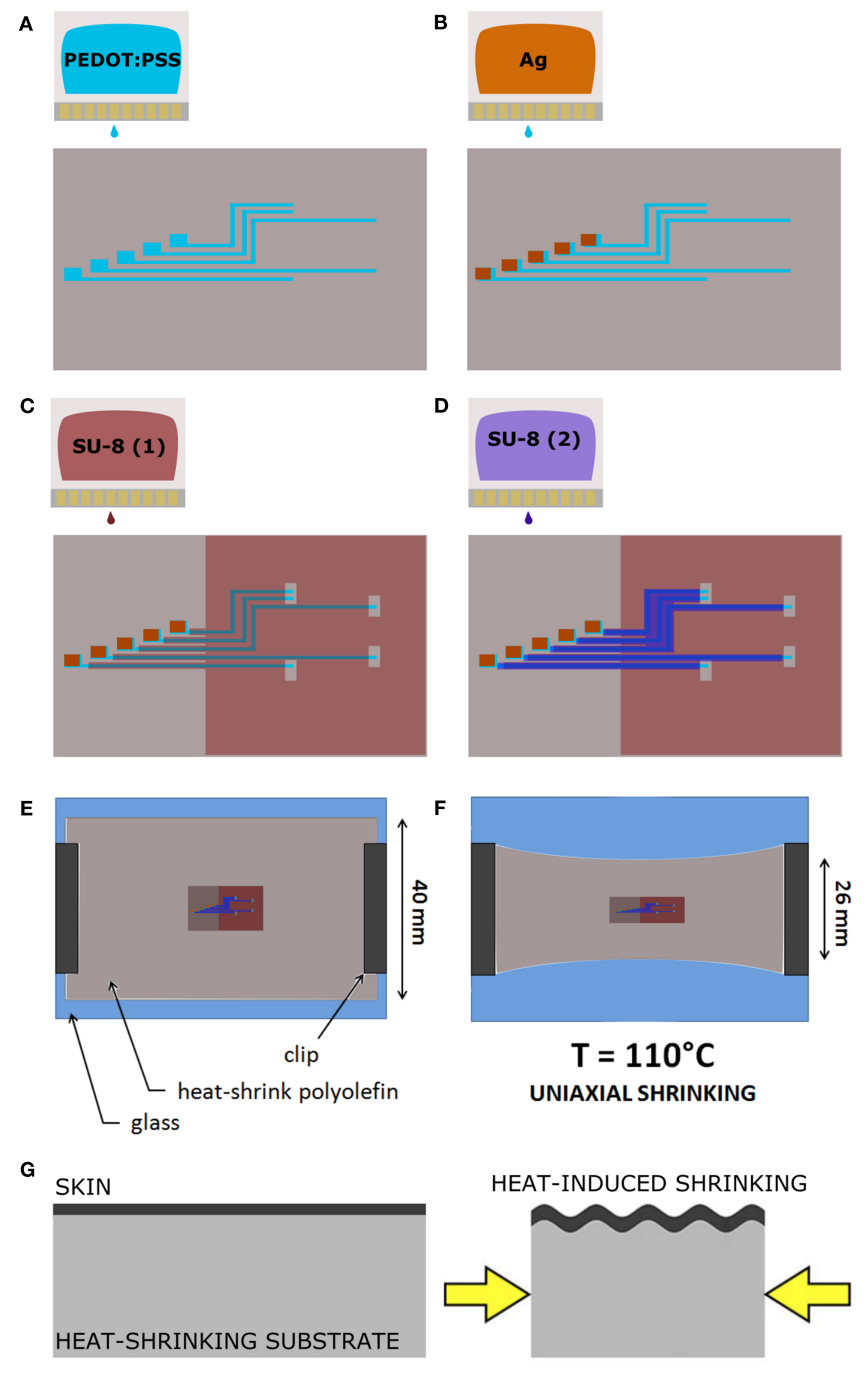
The Print and Shrink fabrication process. **(A)** Inkjet printing of the conductive ink based on PEDOT:PSS. **(B)** Inkjet printing of the Ag ink. **(C)** Inkjet printing of the first SU-8 10% layer. **(D)** Inkjet printing of the SU-8 29% layer. **(E)** PO film clamped onto the glass slide. **(F)** Thermally-induced uniaxial shrinking. **(G)** Schematics of surface wrinkling induced by heat-shrinking.

The Print and Shrink fabrication started with the printing process, which entailed multiple layers deposition (Figure 1) with a lab-scale inkjet printer (DMP-2800; Fujifilm). Heat-shrink PO wrap films (Cryovac D-940; Shrink Packaging Sealed Air, 18 μm thickness) were used as substrates. PO films adopted here had a nominal free shrink of 70–75% upon heating at 120°C. The conductive materials used for printing were a PEDOT:PSS-based ink (PJET700; Clevios) mixed with 10% (w/w) glycerol (Farmalabor), and an Ag-precursor ink (Reactive silver ink; Sigma-Aldrich). As dielectric material, a solution of SU-8 (SU-8 2002; MicroChem Corp.), an epoxy resin-based dielectric, was used. The pristine SU-8 2002 (SU-8 29%) is characterized by 29% solid content in cyclopentanone solution. A less viscous SU-8 formulation (SU-8 10%), characterized by 10% solid content, was obtained by adding cyclopentanone (Sigma-Aldrich) to the SU-8 2002. For each step, a single layer was printed, at room temperature, using 1 jetting nozzle and a specific drop spacing (ds). The ds, which corresponds to the distance between centers of two contiguous jetted drops, was tuned in order to deposit the thinnest uniform layer possible. Before the deposition, polyimide (PI) adhesive tape (Kapton®) and metal clips (Double clips 25 mm; Lebez) were used to fix the PO film (70 mm x 40 mm) to a glass support slide (75 × 50 mm). Air plasma (Colibrì plasma system; Gambetti) was performed to activate the PO surface before each printing step with the following settings: 50 s, 10 W before printing of PEDOT:PSS and Ag ink; 100 s, 10 W before SU-8 printing.

**Figure 2:**
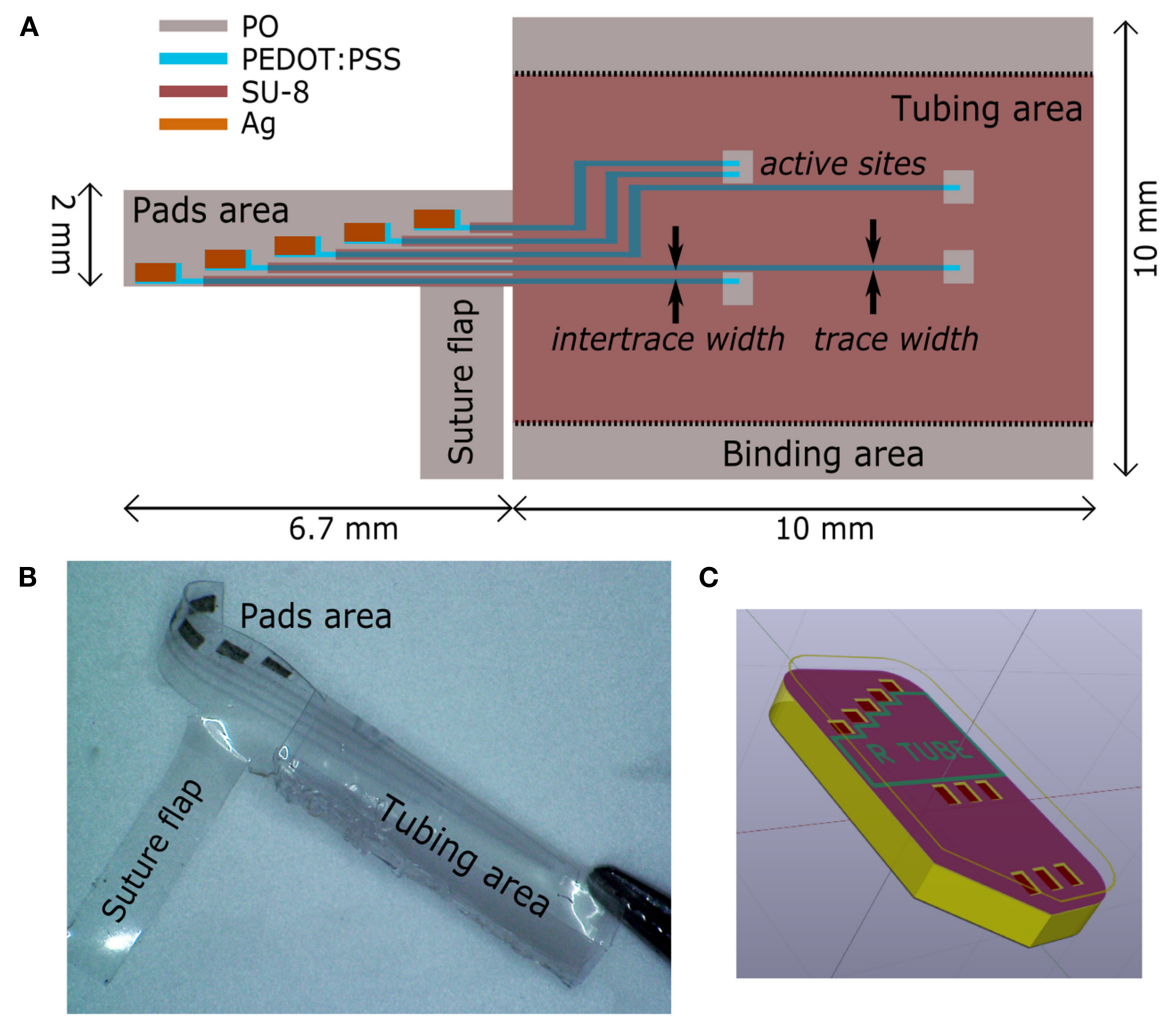
Regenerative nerve Cuff Electrodes (RnCEs). **(A)** Planar view of the final device, with the design of the tube lumen and geometrical references before the rolling up. **(B)** Picture of the final RnCE. **(C)** Rendering of the device with an inset of the custom PCB.

The deposition started with the patterning of the electrodes, the feed lines and the contact pads by printing of the PEDOT:PSS-based ink with 30 μm ds (Figure 1A). Ag ink was then deposited with 40 μm ds onto the PEDOT:PSS terminal pads (Figure 1B). After drying overnight at room temperature, the dielectric layer was printed. Firstly, the SU-8 10% was printed with 70 μm ds [SU-8 (1), Figure 1C], dried by heating (95°C, 60 s), cured for cross-linking by UV exposure (365 nm, 410 mW cm-2, 30 s) with a spot light source (Lightningcure LC8; Hamamatsu) and then heated again (95°C, 60 s). During the thermal processes, in order to avoid its shrinkage, the substrate was accurately fixed to the glass slide by using extra Kapton tape and metal clips, three clips for long sides, one clip for short sides. A layer of SU-8 29% was then printed, with 50 μm ds, to efficiently insulate the conductive traces [SU-8 (2), Figure 1D]. Shortly after the deposition, the tape was removed and the PO was fixed to the glass slide only by clamping the short sides with metal clips (one clip for each). Then, by heating at 110°C for 4 min, the PO film uniaxially shrunk, along the direction parallel to the clamped sides (Figures 1E–G). After shrinkage, the PO was fixed to the glass slide by using Kapton tape and clips on each side (to avoid further shrinkage), and the SU-8 29% layer was cured for cross-linking by UV exposure (30 s) followed by heating (70°C, 4 min). In order to produce the proper final tube's dimension (10 mm length, 2.5 mm lumen) the PO film (≈30 μm final thickness, after shrinkage) was cut as shown in Figure 2A. The “tubing area”, 10 mm square, was rolled up by wrapping it around a cylindrical metal mold with diameter 2.5 mm. The overlapping “binding areas” were bonded by pressing them together with a flat scalpel heated with a hot gun. A thin layer of poly(dimethyl siloxane) (PDMS; Sylgard 184) was added on the closure of the tubes to ensure the sealing. The PDMS was finely brushed onto the closure of the tubes, supported by silanized glass pipettes. The silanization process, adopted to prevent the tubes from sticking to the support, was performed by exposing the glass pipette to 30 min chlorotrimethylsilane (99%, Sigma Aldrich) vapor created in an airtight plastic container by putting a small quantity of solution in a vial (Bernardeschi et al., 2015). The sealed tubes were then left to dry in open air for 1 day.

The final RnCE is depicted in Figure 2B. A custom PCB (CadLine) (Figure 2C) was connected to the RnCE pads area with conductive epoxy (Ag/epoxy adhesive; Chemtronics). Connections were then insulated with UV-curable glue (Bondic; 365 nm, 410 mW cm-2, 60 s). A suture flap (Figure 2B) was left near the pads area to allow the anchoring of the RnCE at one of the nerve ends during the surgical procedure for implantation.

### RnCE Surface and Electrical Characterization

The thickness of each printed layer was evaluated by printing the inks on a silicon wafer and measuring with a stylus profiler (P-6 stylus profiler; KLA Tencor). The inner wall surface topography was analyzed by means of atomic force microscopy (AFM) in order to have an insight on the spatial periodicity and the height of wrinkles. AFM images were acquired with a Veeco Innova Scanning Probe Microscope operating in tapping mode with a silicon probe (NSG01; NT-MDT), and scanning in the direction perpendicular to the wrinkle axis. The images were then analyzed with a free and open source software (Gwyddion^1^) by extracting the sequences of peaks (x-z profiles) along the direction perpendicular to the wrinkles (x axis). To describe the spatial periodicity, the wrinkle wavelength (λ) was defined as the average distance between first-neighboring peaks along the x axis. The wrinkle height (h), i.e., the average of the peak heights, was found by averaging the z axis distances between each peak and the first-neighboring left and right valleys along the profile. Scanning electron micrographs were acquired by using a EVO MA10 SEM (Zeiss), operating at 5 kV accelerating voltage. The electrical characterization of the printed PEDOT:PSS traces was performed with a four point probe tester (Cascade PM5), averaged over four samples.

### *In vivo* Implantation

First, we tested the main features of the RnCEs, their design and the active materials, producing the PPP tubes (Supplementary Material, PEDOT:PSS coated PO tubes fabrication and Supplementary Figure 1). In order to assess whether PPP tubes are able to support nerve regeneration, they were implanted as nerve conduit to bridge a gap in the transected sciatic nerve of rats (Valero-Cabré et al., 2001) and compared with standard silicone tubes. For the implant, female Sprague–Dawley rats (250–300 g) were anesthetized with ketamine/xylacine (90/10 mg/kg i.p.). Then, the sciatic nerve was exposed at the midthigh following a muscle splitting incision. The sciatic nerve was transected 90 mm proximal to the third toe, a portion of 5 mm was resected, and the proximal and distal stumps were sutured to both ends of a silicone tube (10 mm long, 2.5 mm i.d., 0.5 mm thick, *n* = 5) or a PPP tube (10 mm long, 2.5 mm i.d., 30 μm thick, *n* = 6) leaving a 8 mm gap between nerve ends (Figure 4). All implanted tubes were filled with saline. After the nerve repair, the wound was closed with silk sutures and anesthesia was reversed with atipamezole hydrochloride (0.2 mg/kg s.c.).

The active RnCEs (*n* = 6) were implanted following a similar procedure (Figure 4C). First, the sciatic nerve was dissected and transected, and the distal nerve was introduced in the tube to test the functionality of the electrode for stimulation (section Electrophysiological Tests). After the tests, a 5 mm portion of the nerve was resected, and the distal and proximal stumps of the nerve were sutured to the tube of the electrode leaving an 8 mm gap. The tubes were filled with saline, the wounds were closed and animals were left to recover. Although six animals received the RnCE, one RnCE was not functional once implanted so the electrode was left to assess regeneration but the animal was not included in the functional evaluation of RnCEs. Then, also in this group, one of the animals died due to anesthesia-associated complications during nerve conduction test after 45 days of implantation.

The experimental procedures were approved by the ethical committee of the Universitat Autònoma de Barcelona in accordance with the European Communities Council Directive 2010/63/EU. Animals were kept on standard laboratory conditions with a light-dark cycle of 12:12 h and *ad libitum* access to food and tap water. All efforts were made to minimize pain and animal discomfort during surgery and treatments.

### Evaluation of Axonal Regeneration

#### Assessment of Motor Reinnervation

Functional reinnervation of target muscles was assessed at 30, 45, 60, and 90 days post-injury (dpi) by motor nerve conduction tests. Briefly, animals were anesthetized and sub-dermal needle electrodes were placed transcutaneously at the sciatic notch for electrical stimulation using single electrical pulses (Synergy Medelec, Viasys HealthCare). The compound muscle action potentials (CMAPs) of tibialis anterior (TA) and plantar interosseus (PL) muscles were recorded using thin needles placed in the muscle belly in monopolar configuration. The reference electrode was placed at the fourth toe and a ground electrode was placed at the knee. The amplitude and latency of the CMAP was measured, and contralateral intact limbs (*n* = 10) were used as control. The rat body temperature was maintained throughout the test with a thermostatic warming flat coil.

#### Assessment of Skin Sensory Reinnervation

The progression of nociceptive reinnervation of the hind paw was assessed by means of pinprick test at 30, 45, 60, and 90 dpi. Animals were gently kept in a cloth with the sole of the injured paw facing upward, and the skin was stimulated with a needle. Five sites of the lateral side of the paw were tested, being each site stimulated three times, and responses were recorded as positive only when clear reaction (as fast withdrawal and vocalization) was triggered by the stimulation (Navarro et al., 1994). The percentage of positive areas per animal was calculated, and contralateral paws were tested as controls.

#### Histology of Regenerated Sciatic Nerve

At the end of functional evaluation (90 dpi), animals were euthanized by an intraperitoneal injection of pentobarbital, perfused with 4% paraformaldehyde in PBS for 30 min and the sciatic nerves were collected. The nerve guides were clipped and sciatic nerves divided in two parts. Proximal halves were stored in cryoprotected solution of PBS-sucrose 30% with azide 0.1% at 4°C before cryosectioning and immunofluorecent processing. Distal halves were post-fixed in 3% glutaraldehyde−3% paraformaldehyde in cacodylate-buffer solution (0.1 m, pH 7.4) at 4°C for epon embedding.

Proximal segments were embedded in OCT gel (Tissue Tek) before cryosectioning. A cryostat (Leica Microsystems, Germany) was used to collect 15 μm thick cross sections. For immunostaining, sections were first hydrated with TBS, blocked for endogenous biotins (E21390, ThermoFisher), and incubated for 90 min at room temperature in a blocking solution (BS, 1% BSA in TBS) containing 0.1% Tween. Sections were then incubated overnight at 4°C in a mixture in BS of NF200 (AB5539, 1:500 Millipore) and S100 (22520, 1:100 DiaSorin) primary antibodies for staining axons and Schwann cells, respectively. Samples were then washed and incubated again for 2 h at room temperature for biotin amplification of the S100 antibody (BA-1000, 1:200 Vector). Samples were washed again and incubated with secondary antibodies Alexa Fluor 594 Goat anti-Chicken (A11042 1:200 Thermofisher) and Alexa Fluor 488 Streptavidin (S11223, 1:200 Thermofisher) diluted in TBS. Finally, sections were washed and cover-slipped with Mowiol containing DAPI (1:10,000, Sigma) for nuclear counterstain. Sections were visualized with an epifluorescence microscope (Eclipse Ni, Nikon) attached to a digital camera (DS-Ri2, Nikon).

To evaluate the microstructure of the nerve, distal segments were post-fixed with osmium tetroxide (2%, 2 h) and dehydrated through ethanol series prior to embedding in epon resin. Semithin 0.5 μm thick sections were stained with toluidine blue. Microscope (Olympus BX51) images were taken and measurement of cross-section area of the distal segment of the sciatic nerve and quantification of the number of myelinated nerve fibers was carried out using Image J software (Del Valle et al., 2018).

### Electrophysiological Tests

Functional evaluation of RnCEs was performed just after implantation (0 dpi) and after 90 dpi. As indicated above, at 0 dpi, the sciatic nerve was sectioned and introduced in the tube portion of the RnCE, and stimulated as with common cuff electrode implants. Afterwards, proximal and distal nerve stumps were sutured to the ends of the RnCE for allowing nerve regeneration. At 90 dpi, electrical stimulation was applied with the RnCE to the regenerated sciatic nerve to assess the stimulation performance of the implanted electrodes. At both timepoints, biphasic current pulses were delivered through each one of the electrodes against a small needle reference electrode placed proximally near the nerve. Increasing current pulses with a width of 10–100 μs and an intensity up to 8 mA (max charge 800 nC) were delivered by a Digitimer DS4 stimulator. The CMAP was recorded from gastrocnemius medialis (GM), TA and PL muscles using small needle electrodes placed in each muscle (Badia et al., 2011). The CMAPs were amplified (P511AC, Grass), band-pass filtered (3 Hz to 3 kHz) and digitized with a Powerlab recording system (PowerLab16SP, ADInstruments) at 20 kHz. The amplitude of each CMAP was measured baseline to peak and normalized to the maximum CMAP amplitude obtained in each experiment by stimulation of the sciatic nerve with a needle electrode. For each electrode, the threshold current of stimulation that elicited 5, 30, and 95% of the maximum CMAP was determined. The electrode with the lowest threshold value in each RnCE (best AS) was used for data analysis. Finally, the selectivity index (SI) was calculated to quantify the specific activation of a single muscle among the set of three muscles (GM, PL, TA) when stimulating from each electrode, as previously described (Veraart et al., 1993) and the maximum SI (SImax) from each electrode for each muscle was used.

### Data Analysis

Data are presented as mean ± standard error of the mean (SEM). Results have been statistically analyzed by using GraphPad Prism 8 (GraphPad Software, USA). Statistical significance for comparison between groups has been analyzed using one-way ANOVA, or mixed effect models followed by Tukey's *post-hoc* tests when required. Statistical significance is considered when *P*-value was < 0.05.

## Results

### RnCEs Fabrication Strategy

The Print and Shrink process consists of four steps: (1) the direct patterning of materials by means of inkjet printing on PO foil, (2) the heat-shrink induced self-assembly miniaturization, (3) the rolling-up of the foil into a tube and (4) the PCB packaging.

The printing process, depicted in Figures 1A–D, defines the microelectrode array (Figure 2A), which consists of 5 electrodes, with pre-shrink size of 200 × 130 μm^2^, and the feed lines. The PEDOT:PSS-based ink, adopted as main conductive material, was formulated with 10% glycerol, enabling an increased conductivity and facilitating the printing process (Garma et al., 2019). A printed Ag pattern is added on top of the PEDOT:PSS terminal pads (i.e., at the external connection interface) to improve the electrical junction with the PCB. SU-8 is used as dielectrics, in two different formulations. SU-8 10% is used to define the electrodes and SU-8 29% to provide the full insulation of the feed lines (SU-8 29%). SU-8 29% was not used for the whole coverage since it tends to delaminate and crack upon PO shrinking, due to its stiffness. The less viscous SU-8 10% ink was therefore adopted to obtain a thin and flexible coverage. Insights on the SU-8 printing and shrinking are provided in Supplementary Material (SU-8 printing process, Supplementary Figure 2).

After printing, the heat-shrinking is performed. By heating above PO glass transition temperature the shrink-wrap film softened and irreversibly shrunk in-plane, as a result of relaxation of pre-stretched polymer chains. As a consequence, the skin layers (PEDOT:PSS and SU-8) were induced to be buckled, upon the compressive planar forces exerted by the substrate (Figures 1E,F). In the actual setup, the printed PO film uniaxially shrunk to 65% of its original lateral size (≈26 mm final width, Figures 1E,F). The final geometrical area of the electrodes is therefore 130 × 130 μm^2^. Notably, after shrinking the PO wrap film remains flexible, optically transparent, puncture resistant and it can be wrapped and rolled-up in tubular conduits resulting in a flexible PNI (Figure 2B). A custom PCB is finally connected to the RnCE pads through a miniaturized package (Figure 2C).

### RnCEs Surface and Electrical Characterization

Surface characterization allowed to analyze the 3D topography of the RnCE lumen. We found two different textures, a wrinkled one and a smooth one, which are essentially related to the composition of printed materials, the printing parameters and the curing processes.

**Figure 3:**
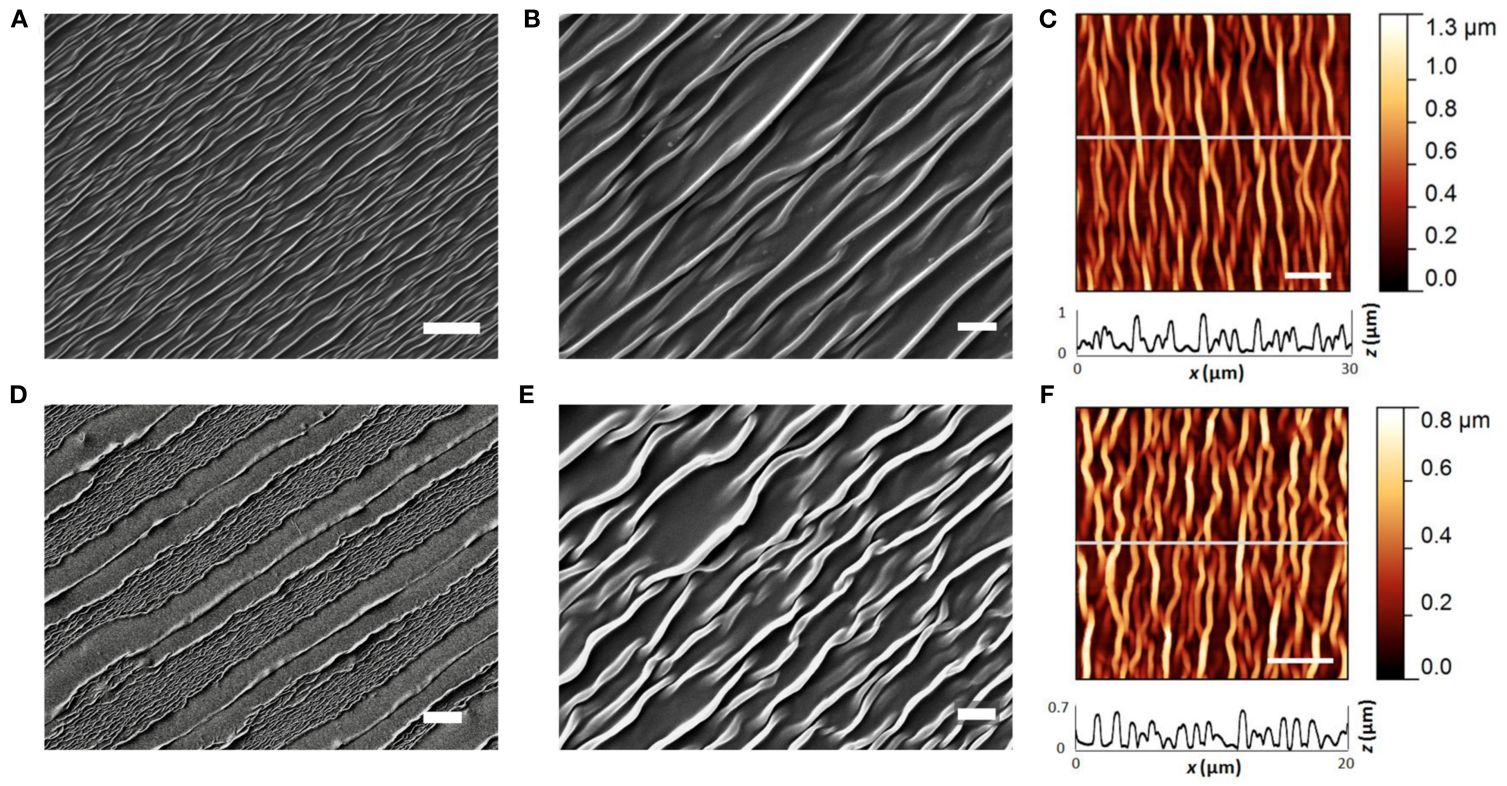
PEDOT:PSS and SU-8 wrinkles. **(A,B)** SEM images of PEDOT:PSS wrinkled active site. Scale bar: 10 and 2 μm. **(C)** AFM image of wrinkled PEDOT:PSS (scale bar: 5 μm), and extracted x-z profile (gray line scan). **(D,E)** SEM images of SU-8 10% wrinkled micro-grooves. Scale bar: 20 and 2 μm. **(F)** AFM image of wrinkled SU-8 10% (scale bar: 5 μm), and extracted x-z profile (gray line scan).

The first texture, which is found on the electrodes and the major dielectric, is related to the wrinkling of PEDOT:PSS and SU-8 10%. Notably, both materials are well adherent to the PO wrap film, since they interlocked with the substrate during its softening. The process ensures the development of compliant interfaces (Supplementary Material, Supplementary Figures 2, 3) without signs of delamination, even after prolonged incubation periods in cell culture medium, as demonstrated in previous studies with a similar heat-shrink substrate (Bonisoli et al., 2017). The PEDOT:PSS film is characterized by an anisotropic (i.e., uniaxial) wrinkled texture, uniform over the whole surface of the electrodes (Figures 3A,B). An average spacing between wrinkles (wrinkle wavelength, λ) of 1.1 ± 0.4 μm, and an average wrinkle height (h) of 0.4 ± 0.2 μm were found (Figure 3C). The SU-8 10% film defined a topography with wrinkled micro-grooves in a periodic pattern with a pitch of around 40 μm (Figure 3D), while the wrinkled areas (Figures 3E,F) showed characteristics very similar to the PEDOT:PSS wrinkled electrodes. Average SU-8 10% wrinkle wavelength was found to be λ = 1.0 ± 0.4 μm and average wrinkle height h = 0.3 ± 0.2 μm. The 40 μm pitch grooves are the result of the self-assembly process in the SU-8 10% layer, where printed lines did not coalesce in a uniform layer. The wrinkling is here confined in the central region of each line, whereas a smooth surface is observed at the edges. The smooth areas did not undergo wrinkling probably because they were too thick due to the so-called coffee ring effect (Hu and Larson, 2006). Nevertheless, nor delamination or cracks were observed in the whole surface. A different texture is observed in the SU-8 29% layer, deposited only on top of the conductive traces. Such dielectric has not been dried neither UV-cured before shrinking. As a consequence, upon PO shrinking, it resulted in a uniform flat coating insulating the underlying bilayer. The coating thickness was 2.7 μm, two orders of magnitude larger than the thickness of the PEDOT:PSS patterns (40 nm). The SU-8 29% cover ensured an adherent interface with PEDOT:PSS wrinkled electrodes (Supplementary Figure 2F).

The electrical resistance of the PEDOT:PSS traces ranged from 144 ± 9 to 249 ± 23 kΩ (average ± standard deviation, averaged over four samples), depending on the length of each trace. Detailed electrical resistance data with respect to traces length are reported in Table 1 (Supplementary Figure 4 for traces reference).

**Table 1:** Electrical characterization of PEDOT:PSS traces, averaged over 4 samples.

**PEDOT:PSS traces**	**Length (mm)**	**Resistance (kΩ)**
1	6.4	144 ± 9
2	7.6	164 ± 26
3	12.6	249 ± 23
4	12.2	214 ± 22
5	9.6	176 ± 28

*See Supplementary Figure 4 for traces reference*.

### Regeneration After Nerve Section and Repair

**Figure 4:**
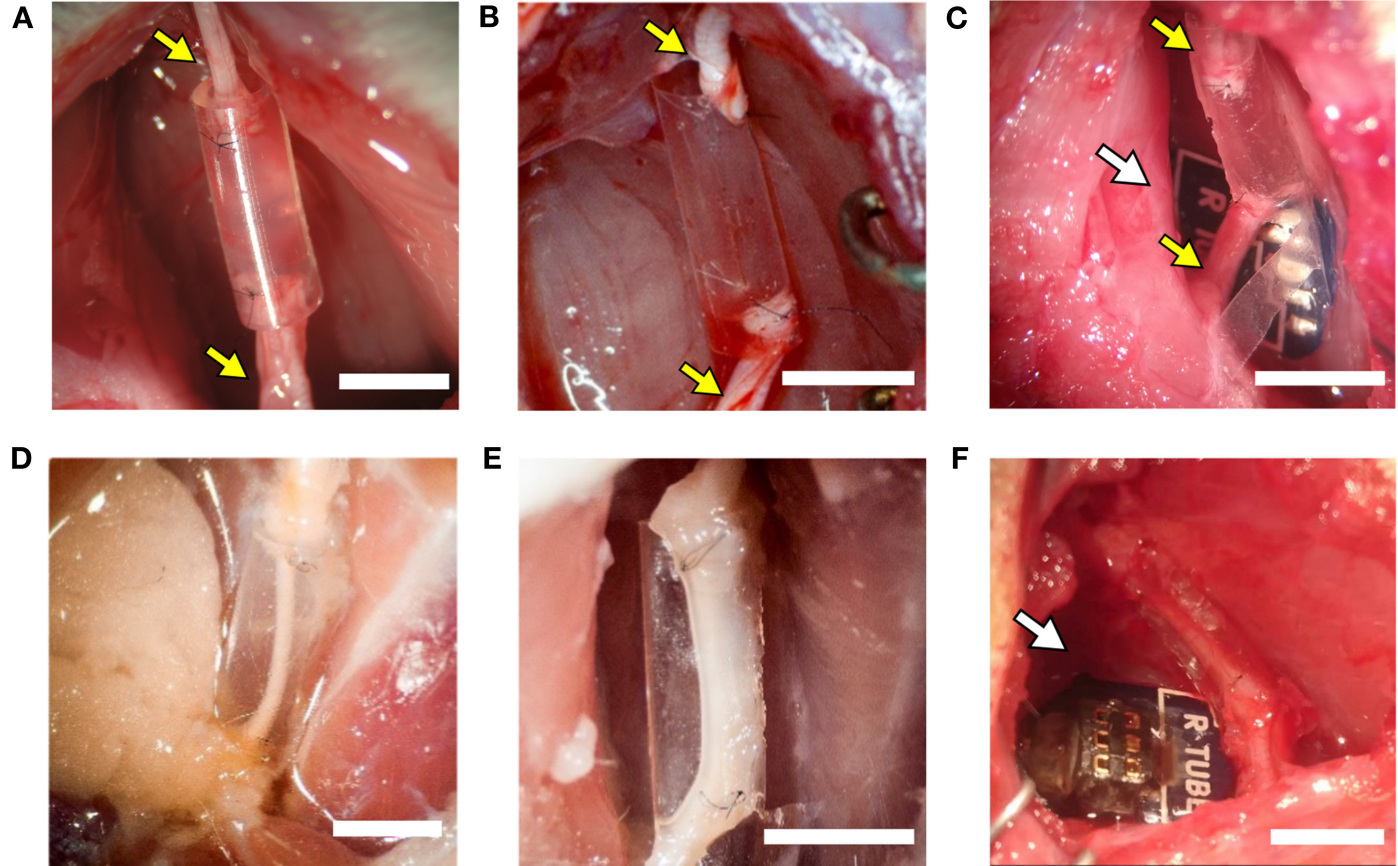
The implanted regenerative cuff electrode. Representative images of: **(A)** silicone tube, **(B)** PEDOT:PSS coated PO tube (PPP tube), and **(C)** Regenerative nerve Cuff Electrode (RnCE) at the time of implant (0 dpi) bridging a 8 mm gap in the rat sciatic nerve, and of **(D)** silicone tube, **(E)** PPP tube and **(F)** RnCE at 90 dpi, showing the regenerated nerve cable inside. Yellow arrows point proximal and distal sciatic stumps sutured to each device. White arrows point the PCB. Scale bar: 5 mm.

**Figure 5:**
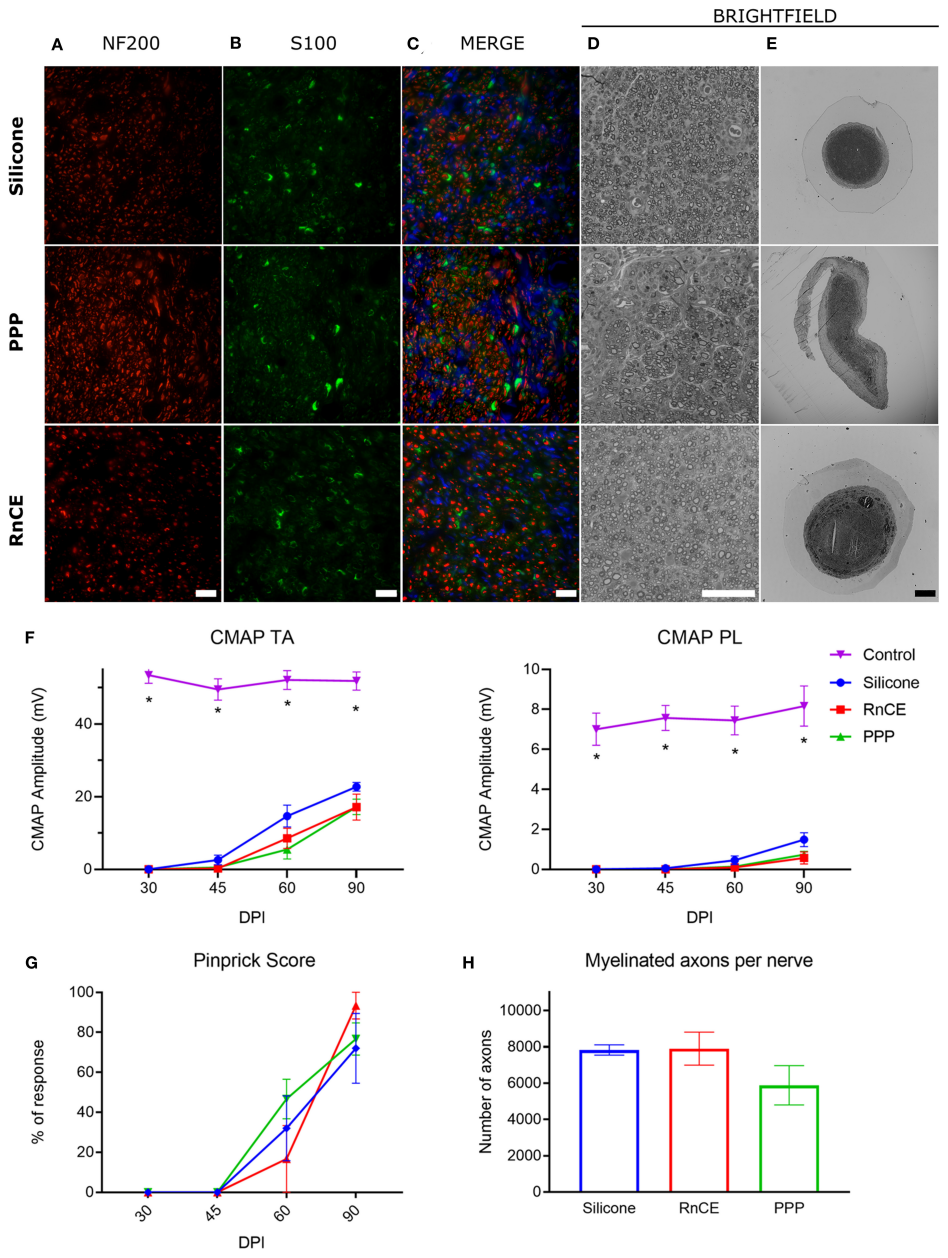
Histological and electrophysiological evaluation of nerve regeneration. **(A–E)** Representative images of cross sectioned nerves repaired with silicone, PEDOT:PSS coated PO tube (PPP) and Regenerative nerve Cuff Electrode (RnCE) at 90 dpi. **(A)** NF200 (red) for axons; **(B)** S100 (green) for Schwann cells; **(C)** Merge of A and B with nuclear staining (DAPI, blue); **(D,E)** Brightfield images of regenerated nerves stained with toluidine blue labeling myelin sheaths at high **(D)** and low **(E)** magnification. Scale bar: 50 μm in **(A–D)**, 200 μm in **(E)**. **(F)** Amplitude of the CMAP of tibialis anterior (TA) and plantar interossei (PL) muscles along the 90 days follow-up after sciatic nerve section and repair. **(G)** Skin paw reinnervation tests by pinprick test. **(H)** Quantification of myelinated fibers in the sciatic nerve distal to the tube in the three tested groups. *p < 0.05 vs. other groups.

Silicone tubes, PPP tubes and RnCEs were compared as conduits to support sciatic nerve regeneration along a 8 mm gap in rats. In all cases, we found a regenerated nerve cable inside the tubes (Figures 4D–F) at 90 dpi. Nerve histological evaluation showed a centered nerve containing a dense core of regenerative units with myelinated and unmyelinated axons and small blood vessels. The immunolabeling of regenerated nerves showed in the three groups a wide distribution of axons surrounded by Schwann cells (Figures 5A–C). Histological sections showed high amount of regenerated nerve fibers without signs of axonal damage, such as no or very thin myelin sheath, or signs of Wallerian degeneration (Figures 5D,E). Quantification of myelinated axons showed no differences (*p* > 0.05, one-way ANOVA) between the three tested tubes (Figure 5H).

We also carried out functional evaluation tests to assess muscle and skin reinnervation following nerve regeneration. Motor nerve conduction tests showed complete denervation of the hindlimb muscles after injury. At 45 dpi, CMAPs of very small amplitude and long latency were recorded from the TA muscle, while responses from the PL muscle, more distal in the paw, started to appear at 60 dpi. All animals showed evidence of reinnervation of TA and PL muscles at 90 dpi. The CMAP amplitude of both TA and PL muscles was significantly lower in the injured side than in the contralateral paw (*p* < 0.05) in all the groups during follow-up, indicating partial reinnervation. Regarding the implanted nerves, the group with a silicone tube had slightly higher CMAP amplitudes of both TA and PL muscles than the other two groups but differences were not significant (p > *0.05*) at any time point (Figure 5F). The pinprick test on the plantar skin showed that pain sensation returned to the paw from 45 dpi, without differences (p > *0.05*) between the three groups (Figure 5G). In summary, functional results indicated motor and sensory reinnervation in animals implanted with RnCEs, comparable to levels obtained with a standard silicone tube.

### *In vivo* Assessment of RnCE Functionality

**Figure 6:**
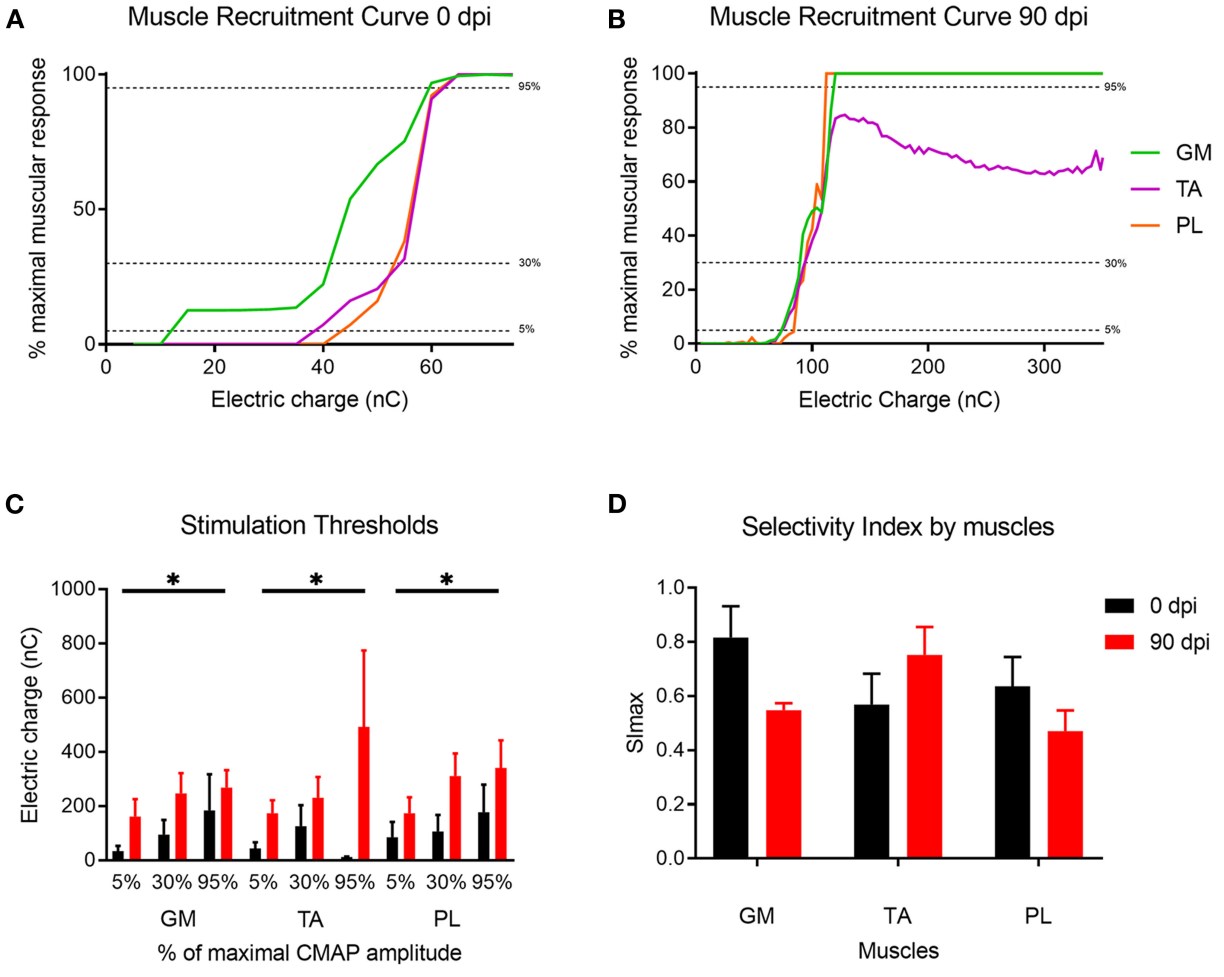
Muscle recruitment curves. **(A,B)** Representative muscle recruitment curves from selected active sites at **(A)** 0 dpi and **(B)** 90 dpi. The increase of the electrical charge delivered through the device elicited recruitment of more muscular fibers thus increasing the CMAP amplitude. **(C)** Values of the electrical charge needed to elicit 5, 30, and 95% of the maximum CMAP amplitude of gastrocnemius (GM), tibialis anterior (TA) and plantar interossei muscles (PL). **(D)** Maximal selectivity index (SImax) for GM, TA, and PL muscles over time. *p < 0.05 vs. time.

The RnCEs were used to stimulate the sectioned but still functional sciatic nerve at the implant time (0 dpi), and then following the same stimulation protocol at 90 dpi on the regenerated nerve (Figure 6). All the RnCEs were able to efficiently stimulate regenerated axons and induce a CMAP in the three tested muscles at 90 dpi. Recruitment curves of muscle activity were plotted for each muscle and compared between 0 and 90 dpi (Figures 6A,B). The thresholds of charge needed to reach a 5, 30, and 95% of the maximal CMAP amplitude significantly increased up to six times after 90 dpi (*p* < 0.05, Mixed-effects) in comparison with values needed at day 0 (Figure 6C). Finally, the capability of the RnCE to selectively stimulate different groups of axons leading to selective recruitment of different muscles was maintained over time, and the SImax showed no significant differences between the acute and chronic implant time-points (*p* > 0.05) (Figure 6D).

## Discussion

In this study we present a novel regenerative PNI, the RnCE, fabricated through a non-conventional low-cost approach, the Print and Shrink strategy. RnCEs connect the microfabrication of CPs, the new frontier in PNIs material, with the development of non-obtrusive regenerative interfaces, the challenge of the next generation PNIs. To the best of our knowledge RnCE is the first fully transparent all-polymer regenerative PNIs.

The Print and Shrink entails low-cost substrate material and cost-effective fabrication techniques. We have already reported inkjet printing as an affordable technique for fabricating all-polymer PEDOT:PSS based multi-electrode arrays on flexible substrates, with application in *in vitro* electrophysiology (Garma et al., 2019). Since inkjet printing does not require any kind of masking, the process enables to save materials and quickly customize the design of the electrodes. Moreover, the optimized printing method here reported allowed the deposition of thin layers, with all the materials adopted, which is crucial to ensure a conformal wrinkling. Indeed, neither cracks nor delamination of all the printed layers were observed after shrinking and the inkjet printed films were stable upon bending of the flexible substrate and remained stable after *in vivo* tests. The wrinkling manufacturing process here reported is an expansion of a previous work on smart multifunctional biointerfaces for muscle and neuronal cells (Greco et al., 2013; Bonisoli et al., 2017). In those cases the self-assembled anisotropic topography and the conductive properties of PEDOT:PSS were found to promote and orient cell growth and differentiation. Here, the texturing of electrodes is meant to increase their surface area while minimizing the electrode size. This strategy has been indeed already reported to enhance charge injection capacity in PNIs (Cogan, 2008). Regarding the biocompatibility of the active material, *in vitro* studies already demonstrated the non-cytotoxicity of PEDOT:PSS coatings in neuronal cells (Charkhkar et al., 2014) and its *in vivo* stability in acute to early-chronic studies (Kozai et al., 2015). Although an exhaustive long-term *in vivo* analysis has not been performed yet, recent studies showed stability of PEDOT:PSS in *in vitro* long-term experiments [one or 3 weeks (Cellot et al., 2016) up to 4 months (Dijk et al., 2020)]. Hence, the facts that our results show a similar amount of axons between standard silicone tubes and the PEDOT:PSS tubes and also that the degree of functional recovery is similar, suggest that this material should be suitable for *in vivo* neural applications.

Remarkably, the here reported low-cost fabrication strategy enables a facile tailorability of the electrodes design. The ease of customization, in terms of electrodes sizing and overall device dimensions, opens for multiple applications with diverse animal models up to personalized peripheral interfaces in case of human lesions. Moreover, the developed technique allows reliable adhesion of the patterned materials, enabling to overcome mechanical instability, as cracks or delamination reported in multiple implants and limiting the neuroprostheses outcome (Barrese et al., 2016; Ganji et al., 2018).

Given their flexibility, RnCEs are suitable for soft interfacing with biological tissues. Nevertheless, the polymeric microelectrode array arranged on the PNI lumen and the whole design do not impair the natural process of nerve regrowth. In this sense, the RnCEs allowed axons to grow through a gap of 8 mm between nerve stumps (Figure 4), a longer distance than used in previous studies with other regenerative interfaces, such as sieve (Lago et al., 2005), microchannel (Srinivasan et al., 2015) or double-aisle (Delgado-Martínez et al., 2017) electrodes. The 8 mm gap chosen is a relatively long distance, but still below the critical length that can be repaired with standard polymer conduits (Lundborg et al., 1982; Valero-Cabré et al., 2001; Yannas and Hill, 2004). We chose the 8 mm gap to prove that both the PPP tube and the RnCE do not hinder regeneration and this material behaves similarly as other plastics such as Teflon (Navarro and Kennedy, 1991) or silicone (Deumens et al., 2010). Moreover, motor and sensory reinnervation outcomes were similar between the silicone, the PPP tube and the RnCE; and the number of myelinated axons did not differ between the three groups. Therefore, our results indicate that the RnCE device (which includes also the PCB that can induce traction forces which could affect regeneration) and the PPP tube alone are as good as conventional silicone tubes in terms of supporting peripheral nerve regeneration. However, the three groups did not reach complete functional recovery after 90 days of implantations, as expected. As with other regenerative electrodes, the RnCE cannot be considered as a better regenerative strategy than other tubulization techniques such as the use of porous materials (Meyer et al., 2016), or fillers with neurotrophic factors (Del Valle et al., 2018) or cells (Allodi et al., 2014), nor better also than the current gold standard as the autograft (Dietzmeyer et al., 2020). Finally, tube or cuff electrodes, such as the RnCE, should be flexible, self-sizing, have a larger diameter than the nerve to be implanted and with the thinnest wall without compromising their stability (Rodri et al., 2000). While potential damage to the regenerated nerve induced by the chronically implanted RnCE is a possibility, the small thickness of the device and the enhanced flexibility of the materials give the tube of the RnCE a soft interface with the nerve.

The RnCE exploits the PNS regeneration capabilities to directly interface the nervous system. In this way the RnCEs overcome the issues related to nerve compression and impaired nerve regeneration, known to affect the interface viability reported for sieve and microchannel devices (Navarro et al., 1996; Wallman et al., 2001; FitzGerald, 2016). RnCEs are able to stimulate regenerated axons, although a higher electrical charge is needed in comparison to axons that had been recently cut. This is attributable to the physiopathological changes of regenerated axons, which are considerable thinner in caliber and with thinner myelin sheaths than normal, for long time after injury (Gómez et al., 1996), and thus have a higher stimulation threshold (Blair and Erlanger, 1933; McNeal, 1976). Moreover, resting membrane hyperpolarization is increased in regenerated motor axons (Moldovan and Krarup, 2004), contributing to the observed lower excitability.

We have characterized the RnCEs in terms of stimulation selectivity, that in PNIs is usually inversely related to the invasiveness of the device implantation (del Valle and Navarro, 2013). Compared with previous studies in the literature, the RnCEs showed a selectivity similar than non-regenerative cuff electrodes (Badia et al., 2011), but lower compared to the double-aisle regenerative electrodes which separated the regenerated nerve fascicles (Delgado-Martínez et al., 2017), or the TIME intrafascicular electrodes applied to the intact rat sciatic nerve (Badia et al., 2011). Hence, RnCEs may not be the best approach for implants in a large nerve trunk, such as the sciatic nerve, but it may be more efficient for implants in smaller nerves, that innervate a limited target. The combination of the technology developed for the RnCE with other regenerative interface designs, such as the double-aisle regenerative (Delgado-Martínez et al., 2017) or the REMI (Garde et al., 2009) electrodes, in which the tube lumen is divided in separate sections by a septum where electrodes can be also located, may be a favorable option to reach higher interface selectivity. In addition, innovative multiple aisle regenerative devices, may allow guidance by molecular cues for sorting the regeneration of specific axonal populations (Del Valle et al., 2018) directing motor or different types of sensory fibers to specific areas of the regenerative interface. RnCEs may also be suitable to be used in cases where nerve transections are surgically repaired with nerve conduits. Interestingly, nerve electrical stimulation with RnCEs might be further exploited to promote axonal regeneration and treat neuropathic pain within the rehabilitation program after nerve repair (Asensio-Pinilla et al., 2009; Cobianchi et al., 2013).

In conclusion, we report here a new fabrication method that can produce a fully transparent regenerative nerve electrode, the RnCE. This electrode can be adjusted in size to house different nerve calibers with a fast and cheap manufacturing process. Future studies will determine whether it has the potential advantages of regenerative interfaces, such as the possibility to record the signals of early regenerating fibers, to generate electrical field to improve regeneration (Gordon, 2016) or to interface axons that have regenerated to bidirectionally connect the subject with an external device (del Valle and Navarro, 2013). Future and more prolonged studies will be aimed at determining whether the RnCE could cause discomfort in the regenerated nerves after more than 3 months, or the materials cause any specific damage in the surrounding tissue. Moreover, it will be investigated the capability of the Print and Shrink strategy to produce microelectrode arrays at higher resolution with increased electrodes density. A significant reduction in electrode size can be obtained by fully exploiting the heat shrinking of the PO substrate. Nominally a bi-dimensional free shrinkage of 70–75% at 120°C can be reached, much higher than the 35% uniaxial shrinkage here obtained. Such improvement will require the setting of a dedicated fabrication methodology to ensure accuracy and repeatability in pattern miniaturization. By maintaining the maximal transparency of the PNI and increasing the microelectrodes array resolution, next generation RnCEs will combine the benefit of good support for axonal regeneration without obstacle or compression with improved selective stimulation and recording capabilities. Finally, the cost-effective Print and Shrink strategy would be useful for the manufacturing of neural interfaces wherein the optical transparency of the device plays a pivotal role, as in the case of optogenetics.

## Data Availability Statement

The datasets presented in this study can be provided by the authors under reasonable request.

## Ethics Statement

The animal study was reviewed and approved by Comissió d'Ètica en l'Experimentació Animal i Humana de la Universitat Autònoma de Barcelona.

## Author Contributions

LMF and AB developed the fabrication strategy (Print and Shrink) under the supervision of FG. AC helped in the design of the electrodes array, developed the PCB, and the electrical interconnections. SM, FG, and XN conceived the study. BR-M and JV performed the *in vivo* experiments under the guide of XN. LMF, AC, AB, BR-M, JV, FG, and XN contributed to the analyses and discussion of the results. All authors contributed to writing and correcting the manuscript.

## Conflict of Interest

The authors declare that the research was conducted in the absence of any commercial or financial relationships that could be construed as a potential conflict of interest.

## Footnotes

^1^https://www.gwyddion.net
