# Supplementary material for: All-Polymer Printed Low-Cost Regenerative Nerve Cuff Electrodes
Source: Front Bioeng Biotechnol. 2021 Feb 10;9:615218. doi: 10.3389/fbioe.2021.615218 (PMC7902501; doi:10.3389/fbioe.2021.615218)
Supplement: Supplementary file 1 [file Data_Sheet_1.docx]

Supplementary Material

S1. PEDOT:PSS coated PO tubes (PPP tubes) fabrication

PPP tubes were produced with a simplified strategy that did not include any patterned electrodes. In this case the PEDOT:PSS-based ink has been spin-coated (2000 rpm, 60 s; Spin150, Semiconductor Production Systems) onto a rectangular (70 mm x 40 mm) PO substrate. After deposition the conductive ink was dried at room temperature for 1 h. The PPP film was then placed onto a glass slide (75 mm x 50 mm) and the two short opposite sides were clamped with one metal clip each. By heating at 110°C for 4 min the PO substrate uniaxially shrunk, along the direction parallel to the clamped sides. The PPP film was then cut into a square shape (10 mm x 10 mm) and rolled-up in a tube by wrapping it around a cylindrical mold with 2.5 mm diameter (Figure S1). The edge areas of the square were bonded by pressing them together with a flat scalpel heated with a hot gun.
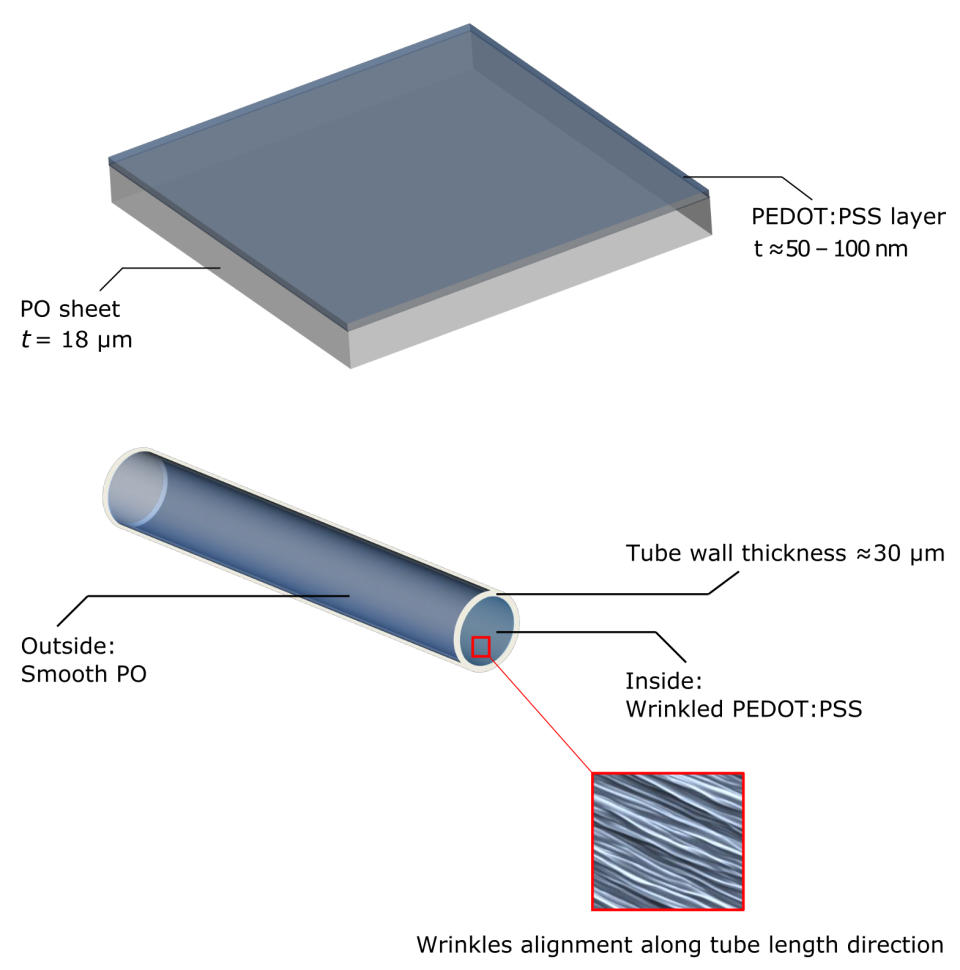


**Figure S1.** Schematic representation of the PPP tubes with their geometrical features both in the PPP film shape (top) and in the PPP tube arrangement (bottom).

S2. SU-8 printing process

Two different behaviours in SU-8 10% printing were observed, by varying the drop spacing (ds). Below a threshold (ds = 50 μm), the PO shrinkage resulted in the delamination of the dielectric layer (Figure S2B). For ds > 50 μm (best results obtained with ds = 70 μm), wrinkles were observed in the central region of each printed line, and a smooth surface at the edges, due to the coffee ring effect (Figure S2C-D). Unfortunately, the coating provided by the SU-8 10% layer was not enough to ensure a perfect insulation of the conductive traces. The SU-8 10% layer was adherent to the wrinkled PEDOT:PSS after PO shrinking (Figure S2C), but some holes were observed on top of PEDOT:PSS traces, probably due to the not complete flatness of the PO surface (Figure S2D). To address this issue, after the deposition of the first dielectric layer and before the shrinking, a SU-8 29% layer was printed in correspondence of the conductive traces. Such SU-8 29% layer was not dried neither UV-cured (before the shrinking), so that, upon thermal shrinking, it could be compressed and resulted in a uniform smooth coating layer. Such coating ensures the insulation of the underlying conductive traces (Figure S2E), and shows an adherent interface with PEDOT:PSS wrinkled electrodes (Figure S2F).
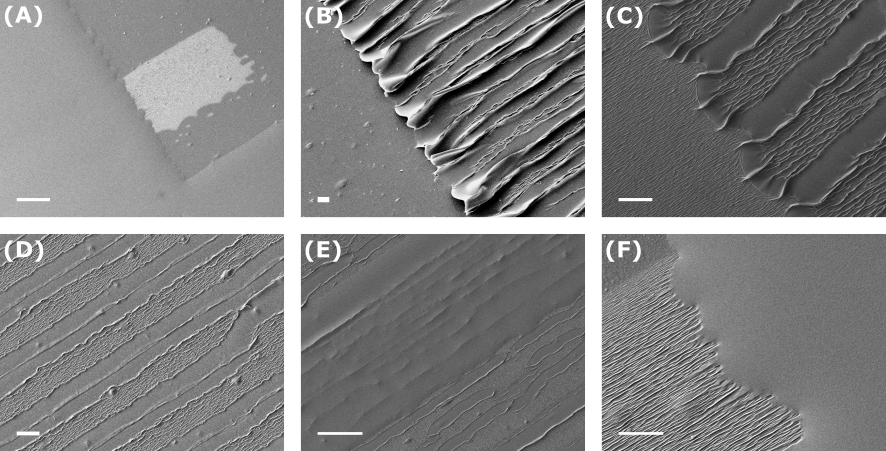


**Figure S2**. SEM images of PEDOT:PSS and SU-8. **(A)** Wrinkled PEDOT:PSS active site and flat SU-8 insulating layer. Scale bar: 100 μm. **(b)** Wrinkled SU-8 10% (ds = 50 μm) on PO substrate. Scale bar: 20 μm. **(C)** Interface between SU-8 10% (ds = 70 μm), with visible double-scale periodicity, and wrinkled PEDOT:PSS. Scale bar: 20 μm. **(D)** Wrinkled SU-8 10% (ds = 70 μm) with flaws due to PO substrate. Scale bar: 20 μm. **(E)** Uniform flat SU-8 29% coating over the bilayer, SU-8 10% and PEDOT:PSS. Scale bar: 100 μm. **(F)** Interface between flat SU-8 coating and wrinkled PEDOT:PSS. Scale bar: 20 μm.


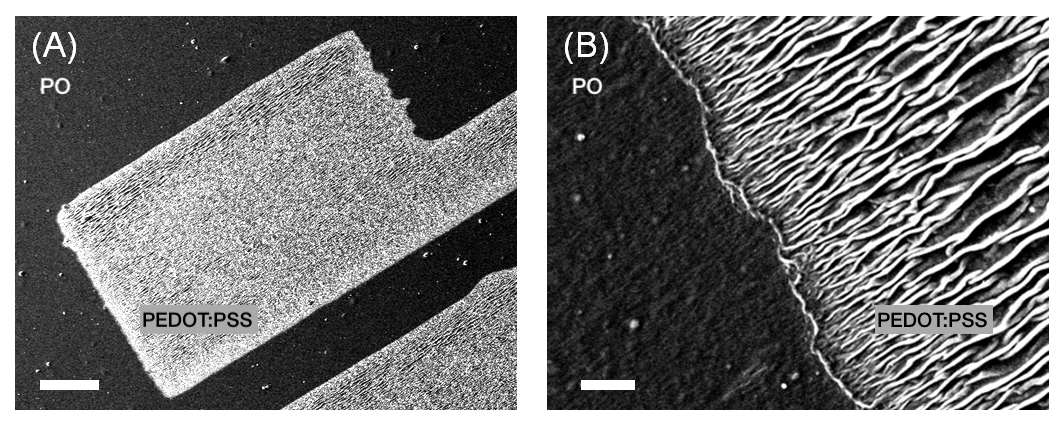


**Figure S3**. **(A)** PEDOT:PSS wrinkled electrode on PO wrap film. Scale bar: 100 μm. **(B)** Interface between wrinkled PEDOT:PSS and the shrunk PO substrate. Scale bar: 5 μm.


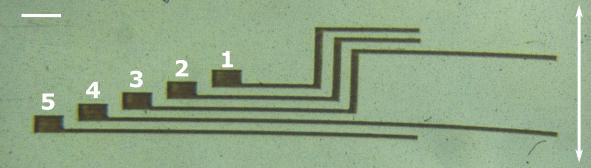


**Figure S4.** Digital microscope image of the microelectrode array after shrinking, with the traces references for the electrical characterisation. White arrow indicates shrinking direction. Scale bar: 1 mm.
